# Disrupted development from head to tail: Pervasive effects of postnatal restricted resources on neurobiological, behavioral, and morphometric outcomes

**DOI:** 10.3389/fnbeh.2022.910056

**Published:** 2022-08-05

**Authors:** Molly H. Kent, Joanna C. Jacob, Gabby Bowen, Janhavi Bhalerao, Stephanie Desinor, Dylan Vavra, Danielle Leserve, Kelly R. Ott, Benjamin Angeles, Michael Martis, Katherine Sciandra, Katherine Gillenwater, Clark Glory, Eli Meisel, Allison Choe, Rene Olivares-Navarrete, Jennifer L. Puetzer, Kelly Lambert

**Affiliations:** ^1^Department of Biology, Virginia Military Institute, Lexington, VA, United States; ^2^Department of Psychology, University of Richmond, Richmond, VA, United States; ^3^Department of Biomedical Engineering, Virginia Commonwealth University, Richmond, VA, United States

**Keywords:** early life stress, postnatal development, bone, lateral habenula, maternal resource deprivation, glucocorticoids, rat

## Abstract

When a maternal rat nurtures her pups, she relies on adequate resources to provide optimal care for her offspring. Accordingly, limited environmental resources may result in atypical maternal care, disrupting various developmental outcomes. In the current study, maternal Long-Evans rats were randomly assigned to either a standard resource (SR) group, provided with four cups of bedding and two paper towels for nesting material or a limited resource (LR) group, provided with a quarter of the bedding and nesting material provided for the SR group. Offspring were monitored at various developmental phases throughout the study. After weaning, pups were housed in same-sex dyads in environments with SRs for continued observations. Subsequent behavioral tests revealed a sex × resource interaction in play behavior on PND 28; specifically, LR reduced play attacks in males while LR increased play attacks in females. A sex × resource interaction was also observed in anxiety-related responses in the open field task with an increase in thigmotaxis in LR females and, in the social interaction task, females exhibited more external rears oriented away from the social target. Focusing on morphological variables, tail length measurements of LR males and females were shorter on PND 9, 16, and 21; however, differences in tail length were no longer present at PND 35. Following the behavioral assessments, animals were perfused at 56 days of age and subsequent immunohistochemical assays indicated increased glucocorticoid receptors in the lateral habenula of LR offspring and higher c-Fos immunoreactivity in the basolateral amygdala of SR offspring. Further, when tail vertebrae and tail tendons were assessed via micro-CT and hydroxyproline assays, results indicated increased trabecular separation, decreased bone volume fraction, and decreased connectivity density in bones, along with reduced collagen concentration in tendons in the LR animals. In sum, although the restricted resources only persisted for a brief duration, the effects appear to be far-reaching and pervasive in this early life stress animal model.

## Introduction

Although the neonatal mammalian brain represents one of nature’s crowning achievements, mammalian neural networks–especially neocortical networks–are far from complete at the time of birth (Rakic, 2009; Jernigan et al., 2011; Noble et al., 2015). The proliferation and migration of glial cells during postnatal brain development, along with the ongoing dendritic arborization and myelination processes necessary to establish optimal neural and glial communication infrastructure, continue during postnatal brain development (Stiles, 2008; Stiles and Jernigan, 2010). Since the 1960s, numerous investigations of enriched and complex environmental influences on postnatal brain development have confirmed the critical role of varied experiences in the development and maintenance of healthy brains (Diamond et al., 1964; Nithianantharajah and Hannan, 2006; Lambert et al., 2016). In addition to CNS development, the skeletal musculature system is critical for regulating animals’ interactions with the environment through movement. Thus, both neural and skeletal-musculature systems contribute to an organism’s adaptive behavioral responses. It has been suggested that the nervous system facilitates an organisms’ interactions with its environment via functions related to both the detection of environmental demands and the execution of movement (Schlinger, 2015). Consequently, the impact of postnatal resources on variables associated with both neural and skeletal systems were of interest in the current study as the far-ranging effects of disrupted environmental resources on biobehavioral outcomes were investigated.

Once the impact of complex environments on developing brains was established in multiple laboratories, research attention focused on identifying neuroplasticity mechanisms contributing to the malleability of brains in varied environments (Fuchs and Flügge, 2014; Sale et al., 2014, 2016). Information about these dynamic neural systems has provided relevant information about effective postnatal interventions for maldeveloped brains due to disease or injury (Brown and Weaver, 2012). Neurodevelopment is also thwarted when the postnatal environment lacks sufficient and appropriate physical or social stimulation (Narducci et al., 2018; Simon et al., 2018; Sylvestre et al., 2018). Because high rates of synaptic growth and restructuring occur during infancy and the early years of childhood, exposure to adverse experiences [e.g., Adverse Childhood Experiences (ACEs)] and inadequate environments (e.g., due to poverty) during this time represents a significant risk factor for the emergence of psychopathological conditions such as depression, addiction, and suicidal ideation (Smith and Pollak, 2020; Oginga et al., 2022). Focusing on the impact of ACEs, for example, repeated exposure to childhood stressors has been associated with compromised brain development in areas implicated in learning abilities and emotional regulatory functions (e.g., hippocampus, prefrontal cortex, frontal-amygdala circuits; anterior cingulate; insular cortex) (Ansell et al., 2012; Hanson et al., 2015; Evans et al., 2016).

Similar to the impact of ACEs, the pervasive effects of poverty, often associated with insufficient social-, material-, and health resources, on neural development present significant threats to developing children. It is estimated that one in three children grow up with some form of multidimensional poverty, with approximately 356 million children across the world exposed to severe poverty conditions (Schmidt et al., 2021). In order to learn more about the specific mechanisms related to the multidimensional aspects of early life stress (ELS), preclinical animal models have been used to examine the roles of neural, endocrine, and behavioral variables (Chen and Baram, 2016; Smith and Pollak, 2020). Maternal separation in rodent models, a preclinical model of ELS, has been used to explore the impact of both acute and chronic maternal separation on developing offspring. Brief separations of dams from their litters via what is sometimes referred to as “handling” stress have been linked to enhanced positive cognitive and emotional effects (Levine, 1957), whereas chronic maternal deprivation (e.g., 24-h separation, or repeated multiple-hour blocks each day) is associated with changes in emotional responses such as increased evidence of anxiety in the elevated plus maze, diminished exploration, and reduced novelty suppressed feeding (Nishi et al., 2014; Miragaia et al., 2018). The maternal separation effects on pups, however, was reported to be inconsistent in varying inbred strains of mice even when the maternal response to pups was consistently heightened upon reunion with pups (Millstein and Holmes, 2007). Alternatively, a simulation of poverty conditions has been investigated in rodents by restricting nesting and bedding resources [known as the limited bedding and nesting (LBN) model], conditions that typically disrupt maternal care and lead to the development of varying neurobiological risks in offspring [e.g., heightened corticosterone levels and adrenal hypertrophy (Avishai-Eliner et al., 2001; reviewed in Molet et al., 2014) and delayed developmental outcomes (Scarola et al., 2020)]. Because this model doesn’t require removing the dam from the nest and the associated feeding and thermoregulation disruptions, the restricted nesting and bedding model of (ELS) offers potential translational value for understanding brain development in adverse conditions (Walker et al., 2017). Regardless of the stress model used, the presentation of psychological stress during the postnatal period is especially disruptive due to the reported dampening of the pups’ natural stress response during this time, a response thought to assure adequate energy for the developing organism by directing valuable resources toward essential development processes (Chen and Baram, 2016).

It is becoming increasingly apparent that the impact of disruptive early life environments extends beyond the developing nervous system. In a study examining the impact of repeated restraint stress and maternal separation in mice (i.e., 6 h/day for PND 0-21), significant reductions in body weight and tail length were observed in the stressed animals, along with delays in eye-opening (Babu et al., 2012; Billakanti et al., 2012). Another study focusing on ELS in mice indicated disrupted bone innervation and metabolism, along with diminished levels of neuropeptide-Y and nerve growth factor, neurochemicals involved in osteoblast differentiation and sensory neural functions in bone, respectively (Wuertz-Kozak et al., 2020). In the same report, disruptions of anabolic bone metabolism were noted in humans following exposure to childhood adversity. These results are interesting considering that bones, like brains, are continuously restructured in response to changing environmental landscapes and demands (Wuertz-Kozak et al., 2020), with multiple neuronal factors serving as neurochemical mediators that also influence bone turnover (Yirmiya et al., 2006; Furuzawa et al., 2014; Jiao et al., 2015). Skeletal effects were also observed in mice exposed to social stress as adolescents; specifically, microcomputed tomography (micro-CT) and histomorphometric analyses indicated reduced length of the tibia and femur bones, as well as altered growth plate thickness and mineral deposition levels (Foertsch et al., 2017). Stress has also been reported to have a negative impact on tendons, a consequential effect considering that tendons serve as a mechanical link between the muscle and bone, and are critical for adaptive movement (Ozturk et al., 2015). Importantly, tendons are known to adapt to changing mechanical environments in a similar fashion as bone tissue (Maganaris et al., 2017). Further, the amalgamation of ELS on multiple neuromusculoskeletal systems is observed in interesting case studies of faltering growth rates in human children experiencing childhood adversity, conditions referred to as psychosocial dwarfism or psychosocial short stature in the clinical literature (Rogol, 2020).

Given the multidimensional effects of ELS (Walker et al., 2017), the goal of the current study was to investigate the impact of a relatively mild simulated poverty model (i.e., moderately restricted bedding and nesting resources) on cognitive, social, emotional, neural, endocrine, morphological, and musculoskeletal variables in male and female rats. Whereas some models incorporate disrupted resources from postnatal days 2–9 (Bolton et al., 2017), we were interested in a more chronic model that would simulate human conditions that are not likely to change during the early childhood developmental period. Accordingly, rats were assigned to either a standard resources (SR) condition during the postpartum period with traditional amounts of bedding and nesting material, or a limited resource (LR) condition consisting of 25% of the bedding and nesting resources provided in the standard housing condition. It was hypothesized that the restricted resource animals would exhibit compromised cognitive responses in the dry land maze (DLM) spatial foraging task, diminished social curiosity in the social investigation task, decreased exploration in the open-field task, as well as altered levels of stress hormones [corticosterone and dehydroepiandrosterone (DHEA)]. Focusing on neural variables, glucocorticoid receptor (GR) and c-Fos immunoreactivity were expected to be increased in LR rats in targeted brain areas associated with increased stress responsivity and heightened threat surveillance, respectively. GRs were of interest due to prior unpublished work in our lab indicating GR-immunoreactivity (IR) in the lateral habenula (LHb); however, to our knowledge, the presence of GR mRNA expression or GR-IR has not been confirmed in the LHb (Aronsson et al., 1988). Following the DLM probe trial intended to enhance uncertainty, increased c-Fos immunoreactivity in the amygdala was investigated as an indicator of responsiveness to the prediction error of reward loss during the probe trial (Kent et al., 2017); further, increased activity in the hippocampus was expected due to its involvement in spatial processing (Ásgeirsdóttir et al., 2020). Related to the musculoskeletal assessments, compromised tendon (i.e., hydroxyproline levels) and bone tissue measures (i.e., trabecular separation, bone volume fraction), as well as delayed growth rates (i.e., body weight and tail length) were expected in the restricted resource (i.e., LR) conditions. Although sex effects were explored, specific directional effects were not hypothesized.

## Materials and methods

### Animals

**FIGURE 1:**
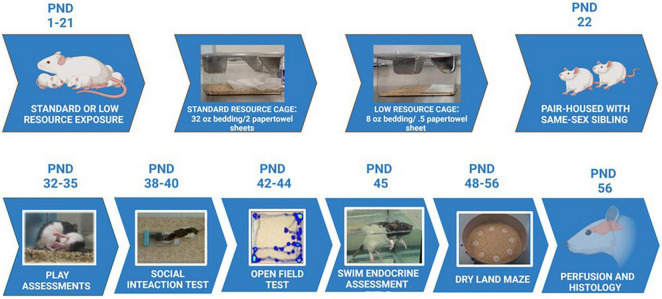
Experimental timeline. Maternal rats were provided with either standard resources (32 oz. bedding and 2 full sheets of paper towels), or LRs (8 oz. bedding and 1/2 sheet paper towel) from PND 1 to PND 21. Maternal behaviors and pup developmental characteristics were measured during this period. Pups were housed with a same-sex sibling starting at PND 22 in standard housing conditions, and assessed for play behavior, social interaction, exploratory behaviors, stress responses, and learning/memory capabilities during the ages of 4 – 7 weeks. Following all behavioral assessments, animals were perfused and brains assessed for immunohistochemical staining, as well as morphological characteristics in the tendons and vertebrae of the tails.

The experimental protocols implemented in the current study were conducted in accordance with the Institutional Animal Care and Use Committee at the University of Richmond. Twelve female (50 day-old) and six male (50 day-old) Long-Evans rats (Envigo Industries, Indianapolis, IN) were pair housed in cages lined with corn cob bedding (Envigo) upon their arrival to the laboratory. Rats were provided rodent chow (Envigo) and water *ad libitum* throughout the experimental period. One week after arrival, a single male was randomly assigned to a cage of pair-housed females. Following three 4-day mating cycles (to maximize likelihood of pregnancy), females were then individually housed in standard laboratory cages until birth. On postnatal day (PND) 1, mothers and pups (litter size ranged from 10 to 13 pups) were moved into one of two randomly assigned treatment groups: a SR group that received 32 oz. of corn cob bedding and 2 full sheets of paper towels for nesting material (*N* = 6 moms), or a restricted low resource (LR) group that received 1/4 the corn cob bedding (8oz.) and 1/2 of a paper towel for nesting material (*N* = 4 moms). After weaning, male and female rats were randomly selected from the mothers in both the standard and LR groups (16 females and 14 males; total *N* = 30; see Supplementary Table 1 for litter size information). Specific offspring group sizes were as follows: SR males (*n* = 6); SR females (*n* = 8); LR males (*n* = 8); and LR females (*n* = 8). At that time, rats were assigned to same-sex dyads, in most cases with a littermate, and placed in standard laboratory cages (48 × 26 × 21 cm) with standard bedding, continued with a 12 h:12 h light-dark schedule (with lights on at 8:00 AM), and were provided with *ad libitum* access to food and water. All cages were changed weekly throughout the study. The resource manipulation continued through PND 21 (see Figure 1 for experimental timeline). Throughout the study, behavioral testing commenced at 9:00 AM.

#### Developmental measurements

Developmental measurements were taken on PNDs 1, 5, 9, 16, 21, and 28 and, in these cases, data are reported as litter averages. Measurements included body weight, tail length (caudal vertebrae length), hindfoot length (calcaneaus to toe tip, excluding nail), eye-opening, presence of teeth, and overall body condition (e.g., milk band, skin condition). For these measurements, sex was not included as a variable since the rats were not assessed for sex and marked individually (with tail marker) immediately after birth to limit handling during this sensitive period. See Figure 1 for the experimental timeline.

### Handling

Starting on PND 28, body weight and other measurements were recorded on a weekly basis throughout the duration of the study. After measurements were recorded, rats received a food reward (i.e., a piece of Froot Loop^®^ sweet cereal). The same food reward was used as an incentive during behavioral testing.

### Behavioral assessments

#### Maternal responsiveness

On PND 9, four pups (two males and two females) were removed from their home cage and placed randomly into a novel cage. The mother was introduced to the novel cage and given 360 s to retrieve and huddle over the pups. The latency to retrieve pups, total time to complete the retrieval task including huddling over pups, and number of pups collected in 360 s were recorded. After the maternal retrieval task was completed, developmental measurements were taken for all pups.

#### Play behavior

On PND 35, rats were separated from their cage mate for 3 h in a new cage and then reunited in order to observe play responses for 1 h a day over three consecutive days. While the rats engaged in play, frequencies of specific play bouts (e.g., rough and tumble play) were recorded. Rough and tumble interactions were classified by the presence of play initiation (i.e., play attack) or a play attack evasion, as well as pins (Schneider and Koch, 2005). An interrater reliability of 90% was used for observers.

#### Social investigation assessment

On PND 38, rats were subjected to a social investigation test (SIT) in a glass tank (76 × 32 × 31 cm) with a thin layer of corncob bedding (3 cm deep). The SIT was conducted over two phases. The first phase was habituation to the glass tank arena and an empty plastic holding container (19 × 10 × 10 cm). The second phase introduced a novel conspecific of the same sex and age that was placed in the plastic holding container. The holding container had holes that allowed the test animals to detect olfactory cues emitted by the stimulus animal. The same stimulus animal was used for the consecutive testing of two cages (from different groups), after which, the container was cleaned with water. Noldus tracking software (Noldus, Leesburg, VA, United States) recorded distance traveled, as well as frequency and duration of interactions with the container at each phase. Blind observers recorded the frequency of rears (internal and external) and grooming bouts during each phase.

#### Open field task

On PND 42 rats were placed in a plexiglas open-field arena (108 × 108 cm). The floor of the testing arena was covered with a thin layer of corncob bedding since the rats were familiar with the surface covering. The bedding was rearranged after each trial to redistribute odors left by the previous animal. Behaviors were scored automatically or manually in Noldus tracking software (Noldus, Leesburg, VA, United States) as appropriate. A random numbers table was used to determine the order each rat was assessed in the arena. Trials lasted 300 s, during which the duration of freezing, crossing the center of the arena, and rearing responses (internal and external directions) were recorded as well as the duration and frequency of thigmotaxis and total distance traveled (in mm.). All measures were automatically collected and scored by Noldus tracking software; additionally, rearing responses were scored manually by observers within Noldus.

#### Dry land maze

**FIGURE 2:**
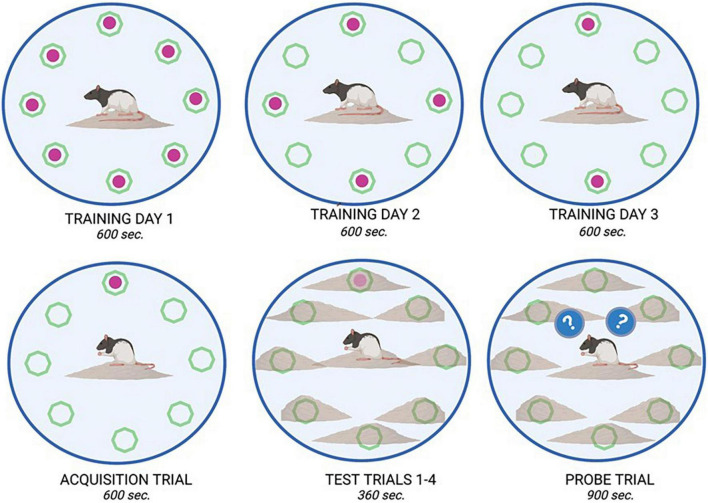
Dry land maze task overview. The dry land maze consisted of an initial training day where animals could retrieve a sweet cereal reward from all available wells (8) within a 600 s time period. On training day 2, only four of the eight wells were baited, and on training day 3, only two of the eight wells were baited. On the fourth day, an acquisition trial was run where only one well was baited with a food reward. This location remained consistent for all animals. On the Test day, four trials were run consecutively, in which the food reward was placed in the same location as in the acquisition trial; however, it was buried beneath bedding to increase the complexity of the test. Animals were allowed 360 s. per trial to retrieve the reward. On the final day, a probe trial (900 s) was run in which no food reward was placed in any well. From this trial, measures such as latency to approach, and time spent in proximity to the previously baited well were recorded as observations of learning and memory.

On PND 51, training for the DLM spatial task consisted of being placed in a 124.5 cm diameter circular arena with 40.5 cm walls (Franssen et al., 2011). The circular arena had eight food wells (2 cm × 1 cm) located equidistant around the periphery. The floor was covered in corncob bedding. To motivate the rats in the DLM task, food was removed from their home cage 3 h prior to each training and testing session. During the first day of training, all eight wells were baited with cereal pieces, on day two of training, four wells (i.e., every other well) were baited, and on the final day of training, two of the previously four baited wells were baited. During the training and testing trials, the empty wells were cleared of any previous cereal treat and fecal boli and the corncob bedding was repositioned between animals (Lambert et al., 2014; Kent et al., 2017). On the fourth day of the DLM, only one of the previously baited wells was baited for the acquisition phase. Throughout the training and acquisition trials, the area around the wells had no corncob bedding surrounding them to increase their visibility; additionally, these trials lasted up to 600 s or until all the cereal treats were consumed. For the next phase of testing trials, the corncob bedding was positioned to cover the food wells so that they would be more difficult to locate visually, requiring the rats to rely on their memory of previous training sessions. The previously baited well was once again baited, and rats were given 360 s to locate the consistently baited well and consume the cereal treat. Once the well was located, the rat was removed from the arena for 60 s and the corn cob bedding was repositioned. Three consecutive trials were conducted in this manner (on PND 55). An observer who was blind to experimental conditions recorded the latency to reach the first baited well and fecal boli emitted during training and acquisition trials. During testing trials, latency to reach the baited well, number of errors made (defined by visiting an un-baited well), and number of fecal boli emitted were recorded. On PND 56, rats were placed in the DLM arena for 900 s for a final assessment, the probe trial. During the probed trial, none of the wells were baited so that rodent responses to the prediction error of not locating the expected reward could be assessed. Observers blind to experimental groups recorded the number of rears (internal and external) and total number of wells visited. See Figure 2 for an experimental diagram of the DLM training and assessment.

### Physiological responses

#### Vaginal cytology

To control for naturally cycling hormones, females were vaginally smeared directly after completing the DLM on all training, test, and probe days. A calcium alginate swab with a diameter of 0.025 cm/0.0635 cm (Puritan Medical Products Company, LLC, Guilford, ME, United States) was first placed in a sterile saline solution and then inserted while the female was gently held on her back. Cells were transferred onto a clean microscope slide and dehydrated with 100% ethanol. The slides were then submerged in hematoxylin stain for 5 min, rinsed, submerged in eosin stain for 5 min, sequentially dehydrated from 50% to 100% ethanol (2 min for each immersion step) before being submerged in citrasolv for 60 s and then coverslipped with Permount (Fisher Scientific, Fair Lawn, NJ, United States). After drying, slides were analyzed at 10X and 20X magnification to determine the estrous cycle. Females in the diestrous stage were tested on the probe phase of the DLM. The diestrous stage was classified as the presence of cornified cells and leucocytes (McLean et al., 2012). To control for handling females for vaginal cytology assessments, males were also gently restrained so the penile area could be lightly touched with a sterile swab.

#### Endocrine responses

To assess stress and resilience responses, the hormones, corticosterone (CORT) and dehydroepiandosterone (DHEA), were extracted from fecal samples. An initial sample was obtained on PND 24, prior to any behavioral testing. To observe stress responsivity, on PND 48 rats were placed in a swim tank (35.744 cm × 17.75 cm × 40.05 cm), filled with water maintained at room temperature (27.94 cm deep; 24°C ± 1°C) for 360 s. A stress fecal sample was collected 12 h after the swim test and at the same time of day as the baseline sample to provide adequate time for the metabolites to move through the digestive system following the swim stress (Bardi et al., 2010; Kent et al., 2017). The collected samples were placed in centrifuge tubes and kept at –80°C until hormones were extracted. The fecal samples were subsequently thawed and 0.09 g of sample was homogenized in 1 ml of 100% methanol. The methanol fecal mixture was then vortexed for 30 s and centrifuged for 15 min. The supernatant was then used to assess CORT and DHEA levels using an ELISA kit (Enzo Life Sciences, Farmingdale, NY, United States). Samples were run on an automated microplate reader (BioTek, Winooski, VT, United States, version 2.04.11) following the provided methods (Enzo Life Sciences) and Gen5 software (BioTek, Winooski, VT, United States, version 2.04.11) to obtain optical density. Readings were assessed at a wavelength of 405λ. The optical density of standards run on the plate were then used to extrapolate the hormone concentrations. Numerical values required an *R*^2^ value greater than 95% to be included in the assessment. The CORT assay had a sensitivity of 27 pg/ml with a range of 32–20,000 pg/ml. The CORT kit had a cross-reactivity of less than one percent for progesterone (0.046%), testosterone (0.31%), tetrahydrocoticosterone (0.28%), aldosterone (0.18%), and cortisol (0.046%). The intra- and inter-assay coefficients of variations for the CORT assays were 7.67 and 9.7%. The DHEA assay sensitivity was 2.9 pg/ml and ranged between 12.21–50,000 pg/ml. The DHEA assay had a cross-reactivity of less than one percent for progesterone (0.06%), testosterone (0.1%), androstenedione (0.73%), and androsterone (0.29%). The intra- and inter-assay coefficients of variations were 5.66 and 7.9% for DHEA.

#### Neurohistology preparations

Sixty minutes after the probe phase of the DLM was completed (necessary for c-Fos activation in response to the probe test), rats were placed in an induction chamber while isoflurane was administered to anesthetize the rat prior to perfusions. Once the animal was no longer responsive, chilled PBS was perfused through the heart at a rate of 40 ml/min using a MasterFlex perfusion pump to clear blood (∼200 ml of PBS) followed by 200 ml of 4% paraformaldehyde to fix the tissue for immunohistochemistry. At this time, the brains were harvested and the carcasses were then stored at –80°C prior to tendon and bone measurements. After 24 h in 4% paraformaldahyde at +4°C, brains were transferred sequentially from a 10% sucrose solution to a 30% sucrose solution. Enough time was permitted between steps for brain tissue to fully absorb the sucrose solution and sink to the bottom of the container. Once brains were cryoprotected, 40 μm free-floating sections were cut on a cryostat (Thermo-Fisher Scientific, Waltham, MA, United States) at –25°C. To prevent double-counting individual cells, every sixth consecutive section was taken for a specific immunohistochemical stain, allowing for at least 200 μm between each section analyzed per immunohistochemical stain.

#### Hydroxyproline analysis

**FIGURE 3:**
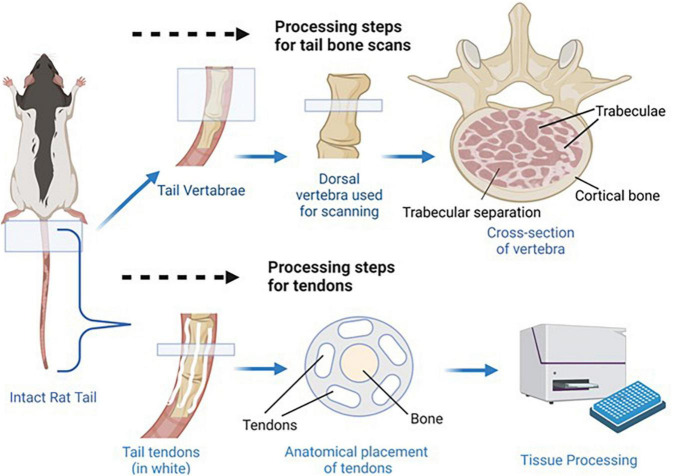
Bone and tendon tissue retrieval. *Shown above*, the most dorsal vertebra of each tail was dissected so that micro CT scans could be used to determine trabecular separation, bone volume fraction, and connectivity density. Anatomical placement of trabecular separation areas, trabeculae, and cortical bone is indicated in the cross section image. *Shown below*, the tendons were dissected from the tails so collagen concentrations could be determined as a measure of tendon health/development. Anatomical placement of tendons and vertebra bone tissue is shown in the tail cross section.

Collagen concentrations of rat tail tendons were determined via a modified hydroxyproline assay as previously reported (Reddy and Enwemeka, 1996; Gouldin et al., 2021). Briefly, tails were thawed in PBS, skinned, and tendons were pulled from the length of the tail. Tendons were blot dried to remove excess water, weighed wet (WW), frozen, lyophilized for 48 h, and weighed dry (WD). The tendons were then digested at 60°C in 1.25 mg/mL papain solution for 16 h, hydrolyzed in 2N sodium hydroxide for 18 h at 110°C, neutralized in hydrochloric acid, oxidized by sodium peroxide, and finally, hydroxyproline content was measured via chromophore produced in 4-(Dimethylamino) benzaldehyde (Sigma-Aldrich) and compared to *trans*-4-hydroxy-L-proline standards (Sigma-Aldrich; Gouldin et al., 2021). Note that for males, due to tissue handling complications, tendon samples were collected from four animals per resource group compared to the eight animals assessed for each of the female resource groups. See Figure 3 for depiction of tail tendon retrieval and processing.

#### Micro-computed tomography

Vertebrae from rat tails were harvested from all experimental conditions and the first caudal vertebra were scanned with a SkyScan micro-CT scanner (Skyscan 1173, Bruker Corporation, Billerica, MA, United States) at high resolution using a voxel size of 11 μm, voltage of 65 kV, and current of 123 μA. The scanning images were reconstructed using NRecon software and 3D constructs were created in CTAn (i.e., a CT analyzer) for morphometric analysis. Regions of Interest were defined at the start and end of the vertebrae. An upper threshold of 255 and a lower threshold of 80 was used to delineate each pixel as “bone” or “non-bone.” The cortical and trabecular bone components were separated manually a few voxels away with an irregular region of interest tool. {Bone volume fraction (calculated as bone volume/total volume, or BV/TV), number of trabeculae in a standardized area [Tb.N. (mm^2^)], distance of trabecular separation in mm, and connectivity density [Conn. Dn. (mm^3^)] were all assessed for tail vertebrae from each animal.}

#### Immunohistology

Following sectioning, brain sections were stained for immunoreactivity to c-Fos, microglia (MG), and glucocorticoid receptor (GR). For MG and GR staining, brains were incubated at room temperature in 0.1% hydrogen peroxide to quench endogenous peroxidase activity. Sections were then blocked for 60 min in 10% normal goat serum (Vector Laboratories, Burlingame, CA, United States) in PBSBT (0.3% Triton-X, Spectrum Chemical, Gardena, CA, United States) and 0.1% bovine serum albumin (BSA; Jackson Immunoresearch Laboratories, West Grove, PA, United States). After blocking, sections were incubated overnight at 4°C with the MG primary antibody, Iba1 [1:10000 (FujiFilm Wako Chemicals, Richmond, VA, United States)] and the GR primary antibody [1:1000 (Signalway Antibody Co, Greenbelt, MD, United States)]. Sections stained for c-Fos were first incubated for 10 min in a 10 mM sodium citrate solution at 100°C water bath for antigen retrieval prior to incubation with hydrogen peroxide. Sections were incubated in primary antibody overnight at 4°C at a dilution of 1:2500 (Immunostar). After incubation in the primary antibody, sections were washed with PBSBT and incubated for 2 h in goat anti-rabbit secondary antibody at a concentration of 1:250 (Vector Laboratories, Burlington, CA, United States). Sections were washed again and incubated in Avidin-Biotin Complex (Vector) for 2 h. Sections were washed again and then incubated in a pre-DAB solution (PBS + 0.6% Tris buffer + 0.3% NH_3_Nis + 0.02% DAB, Vector) and then incubated in DAB (30% H_2_O_2_ + 0.6% Tris buffer + 0.3% NH_3_Nis + 0.02% DAB, Vector). After staining, sections were floated onto subbed slides, dehydrated in sequential ethanol solutions (70–100%), citrasolv, and coverslipped with Permount mounting medium (Electron Microscopy Sciences).

#### Neuroquantification

GR and c-Fos immunoreactivity (IR) were quantified using Neurolucida software [Microbrightfield (MBF), Inc., Williston, VT, United States] and a Zeiss Axioskop light microscope (Carl Zeiss, Oberkochen, Germany). Cell counts were conducted for a 200 × 300 μm^2^ perimeter box at a 40x magnification. Using MBF, GR-IR was quantified in the basolateral amygdala and lateral habenula while c-Fos-IR was quantified in cornu ammonis (CA)1, CA3, and the basolateral amygdala. MG immunoreactivity in the basolateral amygdala (BLA) was quantified using light-thresholding software (Bioquant, Nashville, TN, United States); specifically, a percentage of immunoreactive tissue was calculated based on thresholding the reactive cells in the imaged areas (135 × 135 μm at 40X magnification).

### Statistical analysis

All data were analyzed in SPSS (v.27) and visualized with GraphPad Prism 8. A 2 × 3 (resources × time) repeated measures ANOVA was used to determine the effects of restricted resources on maternal responsiveness. A 2 × 2 × 3 (resource x sex x time) mixed analysis of variance (ANOVA) was used to analyze play data across the three time points. A 2 × 2 ANOVA (resource × sex) was used to analyze the open field behavioral test, social investigation test and DLM probe behavioral data. For DLM training, a 2 × 2 × 4 mixed ANOVA was used. A 2 × 2 × 3 mixed ANOVA (resource × sex × time) was used to analyze the DLM test behavioral data. A 2 × 2 (resource × sex) mixed ANCOVA was used to analyze the hormone data, using an early measure prior to the behavioral tests as the covariate. All neuroquantification data, tendon data, and bone data were analyzed with a 2 × 2 ANOVA (resource × sex). For all analyses, a *P*-value equal to or less than 0.05 was accepted criterion for a significant effect, and individual *post hoc* tests were used when appropriate to determine behavioral effects over time. All non-significant results not described in the text can be found in Supplementary Table 2.

## Results

### Maternal retrieval

For the maternal retrieval tests, a one-way ANOVA was used to assess the effects of restricted resources on maternal responsiveness on PND 9. A trend was observed for latency to retrieve pups (*F*_1_,_7_ = 4.491, *p* = 0.072, $\eta_{\text{p}}^{2}$ = 0.391; specifically, the LR had higher mean values to retrieve all pups compared to the SR group). No significant differences were observed for completion of the task or number of pups collected in 360 s (see Supplementary Table 2 for these results; *p* > 0.05).

### Play behavior

**FIGURE 4:**
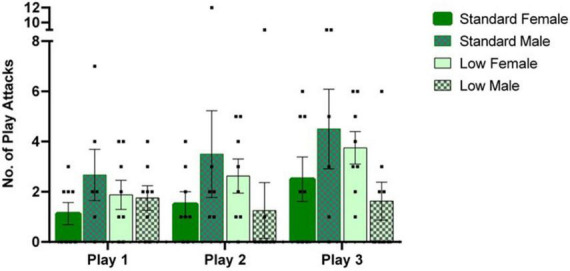
Play behavior task. Play behaviors including rough and tumble play attacks were assessed during a 1-h time window, following a 3-h isolation period. Following a two-way mixed repeated ANOVA, a significant interaction between sex and resources (*p* = 0.04) was observed as well as an effect of time across play sessions (*p* = 0.02). Across, all play sessions, *post hoc* tests revealed that the SR males exhibited a greater number of play attacks than the LR males (*p* = 0.04), with the opposite effect observed in the females (*p* = 0.05). Individual data points overlap bars representing means ± SEM. Standard resource subjects are shown in solid bars, and low resource subjects are shown in patterned bar (*n* = 8 for SR Females, SR Males, and LR females; *n* = 6 for LR males).

A 2 × 2 × 3 repeated measures ANOVA was used to analyze quantified play behaviors. A significant interaction of sex and resource variables (*F*_1,26_ = 4.534, *p* = 0.043, $\eta_{\text{p}}^{2}$ = 0.148) was observed for the number of play attacks exhibited, while a significant effect of time was also observed throughout the assessments (*F*_2,52_ = 4.106, *p* = 0.022, $\eta_{\text{p}}^{2}$ = 0.136). For *post hoc* assessments, individual 2 × 2 ANOVAs and subsequent *t*-tests indicated that the SR males demonstrated a greater number of attacks than LR males, whereas an opposite directional effect was observed in the females (i.e., SR females demonstrated fewer attacks than LR females) across all three trials (*p* < 0.05). Frequency of play attacks increased with subsequent trials (see Figure 4).

### Social investigation task

**FIGURE 5:**
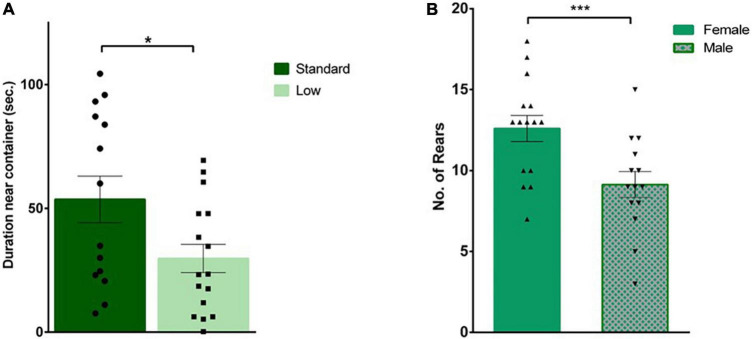
Social interaction task. During habituation, an empty plastic container was placed in the arena for rats to explore prior to the social test. **(A)** Standard-reared rats (•) were found to spend a significantly greater amount of time near the empty container compared to rats raised in a low resource setting (■), (*p = 0.026; *n* = 16 for SR group, *n* = 14 for LR groups). **(B)** During the social interaction trial, when an unfamiliar rat was placed in that plastic container, a significant sex effect was observed where females (▲) exhibited a greater number of external rears compared to male rats (▼) (***p = 0.004). Data represent individual animals over bars showing means ± SEM (*n* = 16 for females, *n* = 14 for males).

A 2 × 2 ANOVA was used to analyze all behaviors during the social investigation task. During habituation, there was a significant effect of resources on time spent with the novel container (*F*_1,25_ = 5.59, *p* = 0.026, $\eta_{\text{p}}^{2}$ = 0.183). Specifically, the SR group spent more time with the container (i.e., a novel object during the habituation trial) compared to the LR group (Figure 5A). When a novel same-sex conspecific was placed inside the plastic container, a significant effect of sex was observed in the frequency of external rears oriented away from the social stimulus (*F*_1,25_ = 9.78, *p* = 0.004, $\eta_{\text{p}}^{2}$ = 0.281), with females rearing significantly more than males (Figure 5B). External rears, total distance traveled, latency to approach container, duration and frequency interacting with same sex conspecific, and grooming were all found to not significantly differ between groups (*p* > 0.05).

### Open field behavior

**FIGURE 6:**
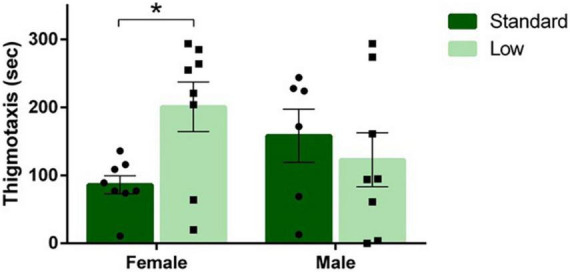
Open field task. Following a two-way ANOVA, a significant interaction of sex and resources was observed for duration of thigmotaxis behavior in the open field task, where females raised in a low resource environment exhibited thigmotaxis longer than females raised in a standard resource environment (*p = 0.035). Individual data points overlay bars showing means ± SEM (*n* = 8 for SR Females, SR Males, and LR females; *n* = 6 for LR males).

A 2 × 2 ANOVA was used to analyze all behaviors quantified during the open field test. A significant sex × resource interaction was observed for amount of time spent near the edge of the arena (thigmotaxis) (*F*_1,26_ = 4.96, *p* = 0.035, $\eta_{\text{p}}^{2}$ = 0.16). LR females spent more time displaying thigmotaxis compared to SR females; an effect not observed between SR and LR males (Figure 6). No other significant differences on freezing, crossing center, rearing, or distance traveled were observed during the open field test (*p* > 0.05).

### Dry land maze

**FIGURE 7:**
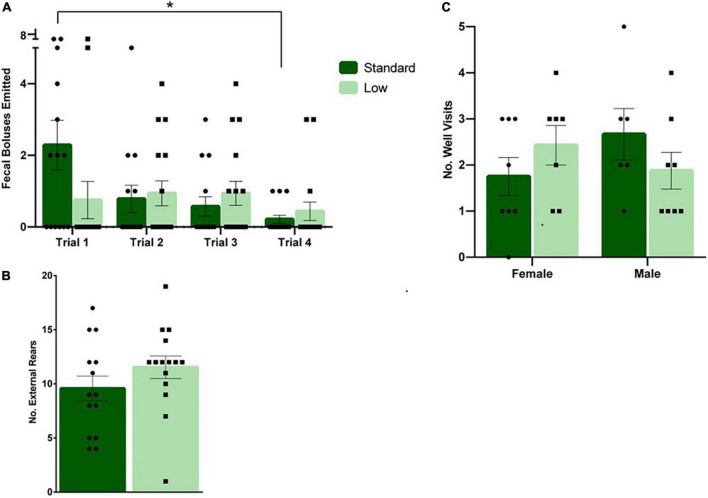
Dry land maze. **(A)** Following a repeated-measures mixed ANOVA analysis on data collected during the four training trials, it was found that standard rats emitted a higher number of fecal boluses during trial 1 compared to trial 4 (*p = 0.031). No differences were detected across time for low resource rats (*n* = 16 for SR group, *n* = 14 for LR group). **(B)** During the probe trial, a trend was observed showing low resource rats exhibited external rears at a greater frequency than standard resource rats (*p* = 0.068; *n* = 8 for SR Females, SR Males, and LR females; *n* = 6 for LR males). **(C)** Also during the probe trial, low resource females were found to visit more wells compared to standard females, and standard males were found to visit more wells than low resource males (*p* = 0.069). Individual data points overlay bars showing means ± SEM; *n* = 16 for SR group, *n* = 14 for LR group.

During DLM training trials, a 2 × 2 × 4 repeated-measures ANOVA was used to determine the effects of sex and resources across training and acquisition trials. A significant interaction of time × resources was observed in the number of fecal boluses emitted during the task (*F*_3,78_ = 3.107, *p* = 0.031, $\eta_{\text{p}}^{2}$ = 0.107). A decrease in the number of fecal boli emitted over time was observed in the SR rats, but no changes were observed over time for LR rats (Figure 7A). No significant differences were observed in latency to the baited wells, number of wells visited, or time to completion (*p* > 0.05). A 2 × 2 × 3 repeated measures ANOVA was used to analyze the differences in groups during the testing stage of the DLM when the food reward was restricted to the consistently-baited well. No significant differences were observed across time or between groups during the testing stage (*p* > 0.05). During the probe test when no food reward was provided, representing a prediction error for the rats, a 2 × 2 ANOVA was used to assess the behaviors during that uncertainty challenge. A non-significant trend for resources was observed for the number of rears toward the wall (*F*_1,24_ = 3.665, *p* = 0.068, $\eta_{\text{p}}^{2}$ = 0.132). The LR group reared toward the wall more than the SR group (Figure 7B). A trend toward an interaction between resources and sex was observed for the number of well visits during the probe test (*F*_1,24_ = 3.634, *p* = 0.069, $\eta_{\text{p}}^{2}$ = 0.132). The SR males visited more wells compared to LR males, and LR females visited more wells compared to the SR females (Figure 7C). No other differences were observed during the probe task (*p* > 0.05). Vaginal smears confirmed that females were in diestrus when they were assessed in the DLM probe test to control for potential endocrine effects.

### Physiological responses

#### Developmental measures

**FIGURE 8:**
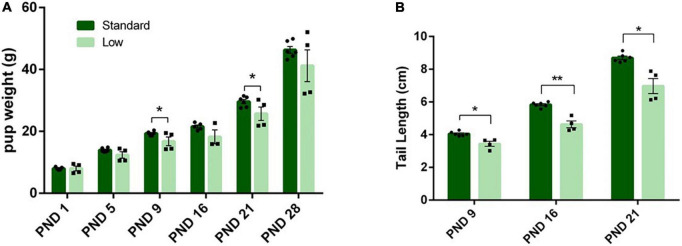
Physical characteristics. Across early postnatal development, a series of measures were collected such as weight gain **(A)**, and tail length **(B)**, among others. **(A)** Pup weight (individual data represented as litter average) is shown to increase across time in both standard- (*N* = 6) and low resource conditions (*N* = 4); however, standard raised pups were significantly heavier at PND 9 and 21(*p = 0.043) compared to low resource pups. **(B)** Tail lengths (shown as litter average) were also significantly shorter in low-resource pups at PND 9 (*p = 0.012), PND 16 (***p = 0.001), and PND 21 (*p = 0.01) compared to standard resource pups. Bars represent means ± SEM with individual litter data points shown in overlay.

On PND 1, 5, 9, 16, 21, and 28, morphological measurements were taken to assess physiological development. No differences were observed between resource groups on eye opening, teeth eruption, or litter size. A 2 × 4 repeated measures ANOVA was conducted on pup weight during lactation, indicating a significant interaction of time and weight (*F*_5,25_ = 3.72, *p* = 0.012, $\eta_{\text{p}}^{2}$ = 0.43). *Post hoc* analysis indicated no differences on PND 1, but a trend on PND 5 (*F*_1,5_ = 6.42, *p* = 0.052, $\eta_{\text{p}}^{2}$ = 0.562) was observed. On PND 9, a significant effect of resources was observed for weight (*F*_1,5_ = 7.31, *p* = 0.043, $\eta_{\text{p}}^{2}$ = 0.594), with the SR group weighing more than the LR group (Figure 8A). On PND 16, no significant effect of resources was observed. On PND 21, a significant effect returned (*F*_1,5_ = 7.1, *p* = 0.045, $\eta_{\text{p}}^{2}$ = 0.586), with the SR group continuing to be heavier than the LR group (Figure 8A). On PND 28, the significant main effect was no longer observed.

A 2 × 3 repeated-measures ANOVA was utilized to assess the length of the tail during lactation (PND 0 – PND 21), using average body weight as a covariate. On PND 16, 21 and 28, a significant interaction of time and resources (*F*_2,14_ = 6.02, *p* = 0.013, $\eta_{\text{p}}^{2}$ = 0.462) was observed; additionally, a significant effect of resources (*F*_1,7_ = 19.62, *p* = 0.003, $\eta_{\text{p}}^{2}$ = 0.737) was detected. *Post hoc* analysis revealed significant differences in tail length at all three measured time points (PND9 *F*_1,7_ = 11.26, *p* = 0.012, $\eta_{\text{p}}^{2}$ = 0.617; PND16, *F*_1,7_ = 27.6, *p* = 0.001, $\eta_{\text{p}}^{2}$ = 0.798; PND21, *F*_1,7_ = 12.32, *p* = 0.01, $\eta_{\text{p}}^{2}$ = 0.638). The LR group had shorter tails compared to the SR group (Figure 8B). Significant differences were not detected between groups for other developmental timepoints such as eye-opeening, hindfoot length, or tooth eruption.: (*p* > 0.05).

#### Endocrine response

A 2 × 2 ANCOVA was conducted on hormone samples, using the initial fecal sample collected prior to the behavioral assessments as a covariate. There were no significant effects of sex or resources on swim-stress CORT fecal metabolite levels. A significant effect of resources was found for DHEA fecal metabolites [*F*(1,25) = 10.87; *p* = 0.003; $\eta_{\text{p}}^{2}$ = 0.303] with LR animals exhibiting higher levels than the SR animals [i.e., *x* = 2951 (+276) and 1447 (+284) for LR and SR groups, respectively] for the low and SR groups following the swim stress. No significant differences were observed in the DHEA:CORT ratio (*p* > 0.05).

#### Hydroxyproline analysis

**FIGURE 9:**
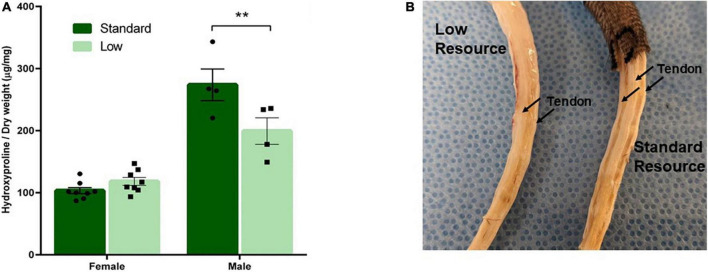
Tendon characterization. One tendon from each tail was removed after PND 56 and analyzed for collagen content. **(A)** Tendon collagen concentration represented by amount of hydroxyproline normalized to dry weight of the tendon and **(B)** representative picture of male low and standard resource tails once skin was removed. Females had no difference in tail tendon collagen concentration between resources at PND 56. Standard resource males had significantly higher collagen concentrations (**p = 0.002) compared to low resource males at PND 56, suggesting more developed tendons. This was also observed in visible assessment with standard resource tails having well defined, larger tendons in comparison to low resource males. Individual data points overlay bars showing means ± SEM (*n* = 8 for SR Females, SR Males, and LR females; *n* = 6 for LR males).

Tail tendon collagen concentration, a measure of tendon maturation, was assessed via a 2 × 2 ANOVA of hydroxyproline content normalized to dry weight on PND 56. A significant interaction of resources and sex was observed (*F*_1,28_ = 12.62, *p* = 0.002, $\eta_{\text{p}}^{2}$ = 0.387). No difference was observed between the female resource groups; however, the SR males had higher concentrations of collagen (hydroxyproline) compared to the LR males, suggesting larger, more developed tendons in standard males. Further, male groups had larger tendons, represented by higher collagen concentrations, than female groups (Figure 9A).

#### Micro-computed tomography

**FIGURE 10:**
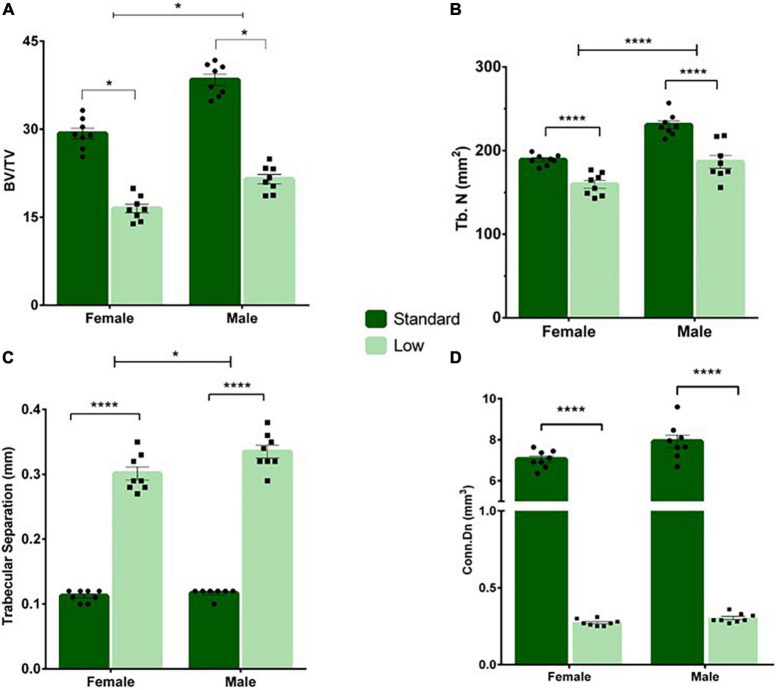
Tail vertebrae characterization and measurements. A variety of morphometric analyses were performed on the tail vertebrae in standard and low resource rats including **(A)** bone volume fraction, **(B)** trabecular number, **(C)** trabecular separation, and **(D)** connectivity density. **(A)** Following two-way ANOVA, a significant sex by resource interaction was found (*p = 0.021) where males had higher bone volume fraction compared to females, and standard rats had a higher bone volume fraction compared to low resource rats. **(B)** A significant main effect of sex was observed for the trabecular number, where the number of trabecula in a given area was greater in males, and in SR rats (****p < 0.001). **(C)** A significant effect of resources was found for trabecular separation (i.e., the distance between trabecula), where LR rats had greater trabecular separation compared to SR rats (****p < 0.001). **(D)** A significant interaction of sex by resources was present for connectivity density, where standard males exhibited higher connectivity density than standard females, and all standard rats had greater connectivity density compared to low resource rats (****p < 0.001). Individual data points overlay bars showing means ± SEM. For **(A–D)**: *n* = 8 for SR females, SR males, and LR females; *n* = 6 for LR males.

**FIGURE 11:**
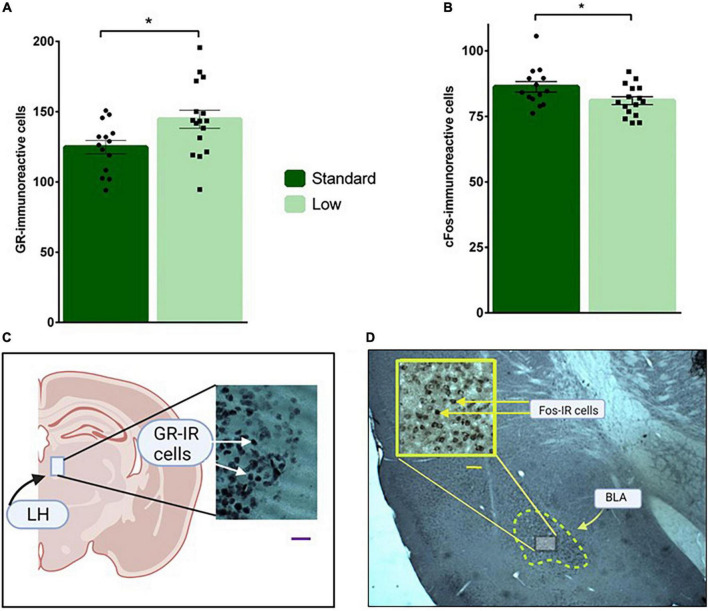
Immunohistochemical analyses. Brain sections were stained for glucocorticoid receptor in the LH **(A)**, and c-Fos immunoreactivity in the BLA **(B)** and analyzed using a two-way ANOVA. **(A)** Low resource rats were found to have increased glucocorticoid receptor immunoreactivity in neurons located in the lateral habenula compared to standard resource rats (*p = 0.032). **(B)** Standard resource rats were found to have increased c-Fos-immunoreactive cells in the basolateral amygdala compared to low resource rats (*p = 0.035). No sex differences were detected for either target of interest. For **(A,B)**: *n* = 16 for SR group; *n* = 14 for LR group. **(C)** Representative photomicrographs of GR-IR cells in the lateral habenula (LH); scale bar = 20 μm. **(D)** Representative photomicrograph of c-Fos-IR cells in the basolateral amygdala (BLA); scale bar = 50 μm. Data are represented as individual data points and bars show means ± SEM. See Supplemental Figure 1 for photomicrographs of negative control for GR- and Fos-IR.

A 2 × 2 ANOVA was used to assess the micro-CT bone scans of animals on PND 56. A significant interaction of resource and sex was observed for bone volume (*F*_1,28_ = 5.934, *p* = 0.021, $\eta_{\text{p}}^{2}$ = 0.175). Males had higher bone volume fraction compared to females, and SR rats had a higher bone volume fraction compared to LR rats, with the SR males exhibiting a greater difference from the LR males than observed between the female resource groups (Figure 10A). A significant main effect of sex was observed for the trabecular number (*F*_1,28_ = 43.29, *p* < 0.001, $\eta_{\text{p}}^{2}$ = 0.61) and a significant main effect of resources was also observed (*F*_1,28_ = 49.83, *p* < 0.001, $\eta_{\text{p}}^{2}$ = 0.64). Specifically, males had significantly higher trabecular numbers (Figure 11B), and the LR group had significantly lower numbers (Figure 10B). For trabecular separation, a significant main effect was observed for resources (*F*_1,28_ = 802.42, *p* < 0.001, $\eta_{\text{p}}^{2}$ = 0.97) and sex (*F*_1,28_ = 5.86, *p* = 0.022, $\eta_{\text{p}}^{2}$ = 0.173). Specifically, the LR groups had greater trabecular separation compared to the SR groups (Figure 10C). A significant main effect of resources was observed for connectivity density [*F*(1,28) = 1758.165, *p* < 0.001, $\eta_{\text{p}}^{2}$ = 0.984] with SR rats exhibiting greater connectivity density than LR rats (Figure 10D). An interaction of resources and sex was also observed for the connectivity density (*F*_1,28_ = 5.85, *p* = 0.022, $\eta_{\text{p}}^{2}$ = 0.173) likely driven by a larger difference in the SR males and females than in the LR animals. where SR males exhibited higher connectivity density than SR females, and all SR rats had greater connectivity density compared to LR rats. No sex effects were observed for LR rats (Figure 10D).

#### Neuroquantification

A 2 × 2 ANOVA was used to assess the immunoreactivity of brains to c-Fos, GR, and MG antibodies. A significant effect of resources on GR-IR was observed in the lateral habenula (*F*_1,26_ = 5.11, *p* = 0.032, $\eta_{\text{p}}^{2}$ = 0.164). Specifically, the LR group had more GR-IR compared to the standard group (Figure 11A). A significant main effect of resources was also observed for C-fos-IR in the basolateral amygdala (*F*_1,26_ = 4.95, *p* = 0.035, $\eta_{\text{p}}^{2}$ = 0.16), with the SR group exhibiting significantly more c-Fos-IR cells than the LRs animals (Figure 11B). Resource by sex non-significant trends were observed for c-Fos-IR in CA1 (*F*_1,26_ = 3.18, *p* = 0.086, $\eta_{\text{p}}^{2}$ = 0.11) and for MG-IR in the basolateral amygdala (*F*_1,26_ = 3.73, *p* = 0.064, $\eta_{\text{p}}^{2}$ = 0.13) These non-significant trends were influenced by SR males exhibiting higher c-Fos-IR levels compared to LR males in CA1. Females, regardless of resources, exhibited a trend for higher c-Fos-IR in CA1. Although not statistically significant, MG-IR in the basolateral amygdala was higher in SR females compared to LR females. LR males also exhibited a trend for higher levels of MG-IR compared to SR males. No differences were detected across groups for GR staining in the basolateral amygdala (*p* > 0.05).

## Discussion

Based on decades of research, the physiological effects of early adverse experiences are considered to be potentially severe and pervasive. In both animal models and humans, negative effects penetrate central and peripheral nervous system functions, leading to dysregulation of cognitive, emotional, immune, and various behavioral systems (Smith and Pollak, 2020). In addition to corroborating previous reports delineating the negative effects of ELS on neurobiological and behavioral outcomes, these findings emphasize the broad reach of these effects to morphometric and musculoskeletal systems. In all, the collective vulnerabilities resulting from a reduction of material resources during postnatal development resulted in altered sex-dependent play, exploration strategies, and performance in the cognitive task (i.e., probe trial). Neural investigations revealed that the LR group exhibited increased GR-IR in the lateral habenula, interpreted as heightened emotional responsiveness. In response to the DLM probe trial, the LR group exhibited less c-Fos-IR in the amygdala, a response interpreted as less sensitive to prediction error associated with the reward removal in the probe trial. Beyond the neurobiological variables, delayed skeletal growth was observed through the first 3 weeks in the LR animals, with lingering indicants of compromised bone and tendon health at PND 56. The convergence of these effects highlights the significant impact of ELS on emotional, cognitive, neural, morphological, and musculoskeletal systems — effects linked to multiple health risks later in life. It is also important to point out that, compared to additional rodent models of early-life stress that incorporate features such as unfamiliar maternal rats and intermittent maternal exposure prompting harmful maternal-offspring interactions, the form of stress in the current study could be considered relatively mild; however, these conditions were continued for a longer period of time than observed in most LBN designs (i.e., 3 vs. 1 week; Walker et al., 2017).

Multiple behavioral assessments were employed to determine the degree to which LBN affects adaptive behavioral outcomes. Play behavior represents a conserved mammalian response during an animal’s juvenile development that facilitates flexible cognitive and behavioral strategies (Auger and Olesen, 2009; Siviy, 2016). Focusing on the frequency of the signature play attacks in juvenile rats, our findings confirmed robust past findings of reliable sex differences in play behavior in rats, namely that males engage in more play than females (VanRyzin et al., 2020). The LBN males and females responded differently, however, with LR females exhibiting more frequent play attacks than their male LR counterparts (see Figure 4). Because a critical role of testicular androgens has been associated with the initiation of play in males, altered patterns of androgen activation could have contributed to the interactions between sex and ELS in these juvenile animals (Cooke and Shukla, 2011), especially considering that the play assessments occurred late in the juvenile period (Bell, 2018). It is also possible that ELS disrupted the development of sex-specific organizational neural patterns since corticolimbic structures are still developing during postnatal development (Bangasser, 2013; Premachandran et al., 2020). Interestingly, social engagement was not affected in the social curiosity assessment, suggesting that the effects of LBN and sex on play responses extend beyond social motivation. Although flexibility in play responses were not specifically assessed in the current design, it would be interesting to incorporate a more sensitive behavioral analysis in future studies to conduct a more thorough analysis of changing response strategies in pups exposed to LBN conditions.

Cognition was assessed using the DLM, a cognitive preclinical assessment task that utilizes positive incentives for memory training to assess foraging efficiency (Kinsley et al., 1999; Kent et al., 2018; Sullivan et al., 2019). Importantly, flexible cognition is observed during the probe trial when a prediction error is introduced (i.e., the expected food reward is no longer in the consistently rewarded location). In the past, complex experiences (e.g., parental experience, training experiences) have been observed to increase efficient and flexible responses in the task (Kinsley and Lambert, 2008; Kent et al., 2018). Due to the known negative impact of stress on hippocampal functions that underlie cognitive processes (Kim et al., 2015), this task which utilizes positive response-outcome contingencies offers value for cognitive assessments. In the current study, results suggested that the LR group was less engaged with the task given that they exhibited increased rear responses against the peripheral wall of the task (associated with escape rather than task engagement; see Figure 7B). Further, during the probe trial, fewer wells were visited by the LR compared to the SR males (interpreted as LR rats being less flexible in response to the prediction error since the other wells were previously baited during training trials), with the inverse observed in females (i.e., LR females exhibited more flexible problem solving based on number of wells visited compared to SR females; see Figure 7C). Further, the SR group decreased fecal bolus emissions throughout the DLM, an effect often associated with diminishing stress levels (Crumeyrolle-Arias et al., 2014). Although no effects of ELS were observed in the number of c-Fos-IR cells in the hippocampus, the finding that the LR animals had fewer c-Fos-positive cells in the amygdala suggests, based on past research indicating positive correlations between basolateral amygdala activation and fear/threat learning (Fanselow and LeDoux, 1999), that the LR animals had a less intense response to the prediction error presented in the probe trial (see Figures 11B,D for c-Fos results). Similarly, in a previous study that exposed pups to a stressed dam during the postnatal period, a sex-specific reduction in brain-derived neurotrophic factor (BDNF) DNA methylation in the amygdala was observed (Roth et al., 2014). Interestingly, a reconceptualization of the amygdala as a mediator of behavioral engagement rather than a specific mediator of fear responses, especially in naturalistic assessments of species-relevant stressors, has been suggested (Paré and Quirk, 2017). Further, although the prediction error neural circuit is viewed as a component of fear processing (McNally et al., 2011), the probe trial used in the current study represents a mild stressor compared to stimuli typically used in fear responses studies. Considering that many psychopathologies in humans are linked to increased avoidance and less engagement with relevant tasks, the LBN model used in the current study, with modest reductions in home cage resources, offers potential for translational research in this area (Schlund and Cataldo, 2010).

Also related to the stress response, the finding of increased GR-IR in the LHb of the LR animals (see Figures 11A,C for GR results) suggests that they developed heightened stress-mediated inhibitory effects, in accordance with past research documenting stress-induced modification of GR levels in limbic areas such as the hippocampus (Ga̧dek-Michalska et al., 2013; Sullivan et al., 2019). The LHb is associated with depressive symptoms, especially related to the inhibition of reward processing (as reviewed by Webster et al., 2021). Additional research tracking the specific area of activation of GR-IR cells (e.g., cytoplasmic vs. nuclear) will be informative in understanding the short- and long-term effects of stress on the LHb GRs (Han et al., 2017). Considering the putative role of the LHb in targeted responses to negative prediction errors, modified GR-IR in this area could lead to distorted reward processing, a condition associated with depression (Proulx et al., 2014). Further, pups exposed to ELS in a previous study exhibited increased LHb cellular excitability and decreased GABA neurotransmission, suggesting that the LHb is sensitive to ELS conditions (Authement et al., 2018). Although prior research failed to detect mRNA expression of GRs in the LHb of male rats (Aronsson et al., 1988), this pervasive receptor is thought to exist throughout all brain areas (Madalena and Lerch, 2017). Interestingly, it is noteworthy that mRNA expression of the Glucocorticoid-Induced Receptor (GIR) has been documented in the LHb (Sah et al., 2005). Thus, additional research assessing GR-IR in the LHb will inform these preliminary findings. Regardless of the role of GRs in ELS-mediated LHb processing, research suggests that this brain area plays a complex role in HPA functions (Jacinto et al., 2017).

In contrast to the neural findings, the endocrine assessments did not suggest that the maternal resource manipulation affected CORT levels in response to swim stress. Although immediate endocrine responses are informative, it would be interesting to track the recovery to baseline values in future studies. Another limitation of the endocrine data is the lack of a true baseline measure. Considering that fecal levels of CORT and DHEA metabolites were obtained prior to the onset of the behavioral assessments, the young age (i.e., adolescence) of the animals at this time was not appropriate for true baseline values; consequently, the first endocrine measures were used as a covariate.

The findings of increased thigmotaxis in LR females in the open field task, an effect not observed in LR males, suggests that females may experience more LBN-dependent anxiety than males (see Figure 6). This finding is aligned with human research suggesting that women are more vulnerable to the maladaptive effects of ELS; further, women are twice as likely to be diagnosed with stress-related psychopathologies such as major depression disorder (Kessler, 2003; Kendler and Gardner, 2014; Reynolds et al., 2015; Goodwill et al., 2019). Interestingly, the higher number of fecal boluses emitted during the DLM training trials observed in the SR animals, regardless of sex, suggests that the ELS-induced anxiety responses were situation specific in the current study (see Figure 7A for fecal bolus data).

Differential attention of the dams toward male and female pups could contribute to the behavioral sex differences observed in the current study. Previous research indicates that dams exposed to restricted resources respond to male pups with less adverse responses (e.g., dropping, stepping on pups) and more nurturing responses (e.g., increased grooming) than directed toward female pups (Keller et al., 2019). Male preferences have been observed in previous studies when dams are reunited with their pups following a period of separation (Orso et al., 2019). Further research focused on sex-dependent maternal care in ELS models is necessary to fully understand the mechanisms of disrupted maternal care effects on offspring.

The most extreme effects of LBN exposure in the current study were observed in the bone and tendon data. Intrigued by casual observations of shorter tails in ELS animals in previous unpublished studies in our laboratory, morphological (body weight, tail length) and skeletal measures (connectivity density and trabecular separation of tail vertebrae) were systematically collected in the current investigation. Additionally, the tail tendon maturation was also assessed (e.g., hydroxyproline concentration). Notably, the LR rat tail vertebrae exhibited approximately half the bone volume fraction as their SR counterparts, with a nearly 3-fold increase in trabecular separation resulting in an approximate 96% decrease in trabecular connectivity, characteristics associated with higher risk of fracture in humans (Chen et al., 2013; see Figures 10A–D). Added to this vulnerability, tail tendon collagen, represented by levels of hydroxyproline, was reduced by approximately 30% in male LR rats (see Figure 9). Collagen is the major component of tendons and provides all of the strength and function in tendons. Lower concentrations of collagen in the tendon suggests reduced development or maturation, which could lead to reduced function and tendinopathy. Due to the role of tendons in the transfer of muscle force to the appropriate skeletal structure, a reduction of tendon maturation represents a significant threat to the musculoskeletal system (Snedeker and Foolen, 2017; Zabrzyński et al., 2018, 2020). Although caution should be taken in the evaluation of the tendon data in the current study due to modest group n’s, the reported effect sizes are suggestive of an influence of ELS on this variable. Further, it is noteworthy that the tail length differences were no longer observed at PND 35 (see Figure 8B), yet the bone and tendon effects were observed at PND 56. The impact of these musculoskeletal effects on rats’ survival in natural habitats is unknown due to the scarcity of information about the adaptive functions of rodent tails. Current hypotheses suggest that rat tails are associated with enhanced balance; additionally, recent research has focused on the role of tails in thermoregulation, with a behavior known as tail-belting recently described as a method wild rodents use to warm their tails by wrapping them around their bodies (Owens et al., 2002; Stryjek et al., 2021).

The pervasiveness of LBN-related musculoskeletal effects throughout other skeletal systems through the body are unknown at this time. In addition to shorter tails observed in LR animals, preliminary observations of shorter foot lengths in LR rats were observed in the current study, an observation that deserves further investigation. It is also important to note that, while stress and anxiety have been shown to negatively affect musculoskeletal tissue health and maturation (Ozturk et al., 2015; Foertsch et al., 2017; Wuertz-Kozak et al., 2020), musculoskeletal development is also well documented to be driven by mechanical stimulations, with reduction or removal of mechanical cues during development–resulting in underdeveloped musculoskeletal tissues (Connizzo et al., 2013; Galloway et al., 2013; Andarawis-Puri et al., 2015; Arvind and Huang, 2017). Therefore, it is difficult to separate whether the differences observed in musculoskeletal development in this study are attributable to varying neurobiological responses or mechanical loading environments, due to differences in bedding, mobility, body weight, or degree of play. Interestingly, while LR males exhibited reduced tendon development at PND 56, females exhibited no difference in tendon collagen concentration due to their resource group assignment. It may be that, since females raised on low bedding play and rear more, they engage in more physical activity involving tail use, which in turn stimulates tendon maturation enough to overcome the decreased musculoskeletal development observed in male LR rats. Alternatively, it may be that estrogen and other hormones produced during estrus drive increased collagen production to compensate for altered levels in LR female tendons (Hansen and Kjaer, 2016; Chidi-Ogbolu and Baar, 2018). However, interestingly, bone health is still significantly reduced in LR females. Thus, similar to the broad impact of ELS-driven neural effects on maladaptive behavioral responses, LBN-driven musculoskeletal effects also influence the efficiency and precision of behavioral responses, presenting further risk of adverse health conditions.

Taken together, these results confirm previous reports indicating a negative impact of adverse conditions during postnatal development in the developing rats (Luby et al., 2020). Past research has indicated the role of heightened hypothalamic-pituitary-adrenal (HPA) axis activity in ELS conditions. Increased levels of stress exposure during the stress noted hyporesponsive period extending through the first 2 weeks of rat postnatal development likely have long-lasting effects on subsequent HPA functions (Lai and Huang, 2011; Maniam et al., 2014). It is also possible that the HPA axis is programmed in sex-specific patterns that underlie observed sex-specific vulnerabilities for psychiatric illnesses related to anxiety and depression (Bangasser, 2013). The trend toward less efficient maternal retrieval responses observed in the LR dams in this study suggests that the current model compromises maternal care of offspring, a response linked to heightened offspring stress and negative health outcomes (Curley and Champagne, 2016). Increased glucocorticoid receptors observed in the LR rats’ LHb, regardless of sex, provide further evidence of HPA involvement. The LHb, known for its role in mediating midbrain aminergic structures with forebrain limbic areas, is increasingly being investigated as a potential neural hub for the emergence of major depression disorder (Browne et al., 2018). Viewed as a “disappointment center,” the LHb is thought to play a role in anhedonia via misinterpreting rewards as punishment-related signals (Shabel et al., 2019). Thus, increased GRs may heighten the sensitivity of this important emotional regulatory hub and tilt it in the direction of life-long susceptibility to depression symptomology. Interestingly, we observed GR-IR staining in the medial habenula as well and plan to investigate its role in offspring development and emotional responses in the future. A clear limitation of the current study was the focus on such a few brain areas; additional research focusing on effects in brain areas implicated in stress responsivity and emotional regulation will inform the current findings.

In addition to the putative role of HPA activity in the emergence of maladaptive responses in LBN rats, it’s also important to consider the physiological impact of the differential physical environments in the two experimental groups. Serving as a primary mechanosensitive organ, the developing bones of the SR and LR rats experienced varying biomechanical pressure in the different environments (Gerosa and Lombardi, 2021). Rich interconnections exist between bone and brain, including the endocrine functions of the skeletal system (Zofkova, 2015), as well as serotonin and glutamate signaling in osteocytes (Warden et al., 2005; Brakspear and Mason, 2012). The potential impact of bone modifications in the LR condition deserves further investigation to elucidate the reciprocal interactions between the nervous and skeletal systems, two systems that are essential for the production of movement and behavior in response to changing environmental conditions. Thus, the restricted resource model of ELS (i.e., the LBN model) used in the current study provides valuable opportunities to explore the mechanisms associated with adaptive and maladaptive behavioral responses to disruptions in physical and social postnatal contexts. Considering the pervasive effects observed in the current study, it is important to consider the reciprocal interactions between the nervous system and other physiological systems (e.g., musculoskeletal system) contributing to adaptive responses in future investigations.

## Data availability statement

The data supporting the conclusions of this article are available from the corresponding author on reasonable request.

## Ethics statement

The animal study was reviewed and approved by Institutional Animal Care and Use Committee (University of Richmond).

## Author contributions

MK, RO-N, JP, and KL contributed to developing the study, collecting data, data analysis, and writing the manuscript. JJ contributed to data analysis and writing the manuscript. GB, JB, SD, DV, DL, KO, BA, MM, KS, KG, CG, EM, and AC contributed to data collection. All authors contributed to the article and approved the submitted version.
