# Supplementary figures and images for: Disrupted development from head to tail: Pervasive effects of postnatal restricted resources on neurobiological, behavioral, and morphometric outcomes
Source: Front Behav Neurosci. 2022 Aug 5;16:910056. doi: 10.3389/fnbeh.2022.910056 (PMC9389412; doi:10.3389/fnbeh.2022.910056)

# Negative control images for ICC

**10X (cFos in BLA)**

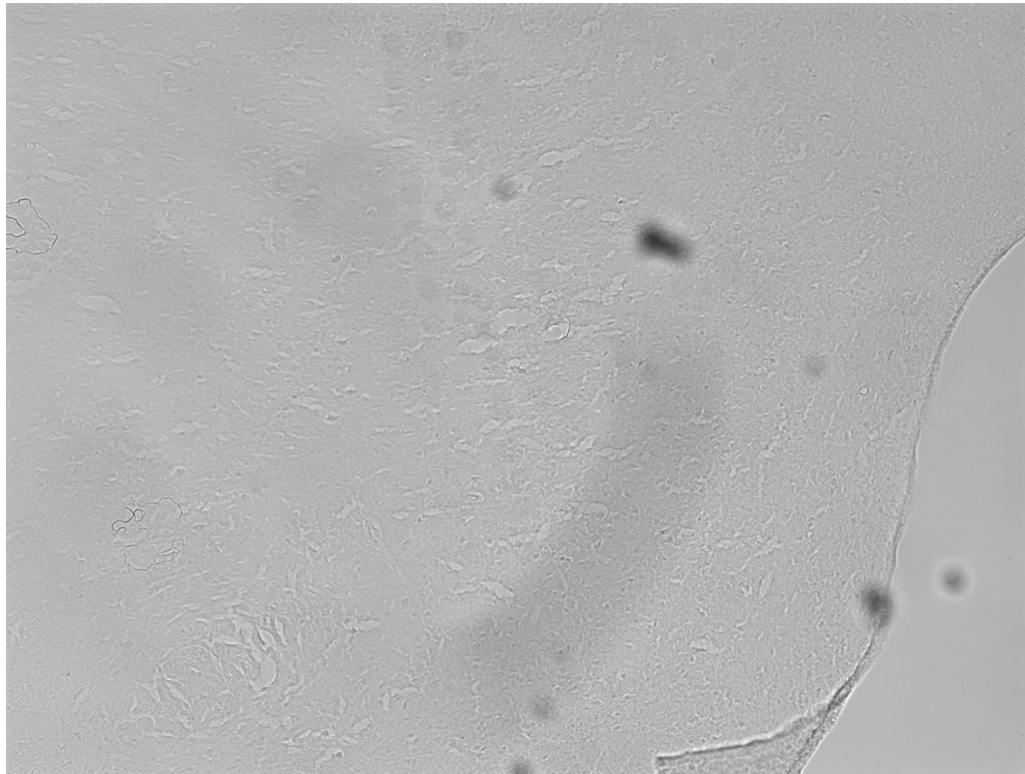

**10X (GR in habenula)**

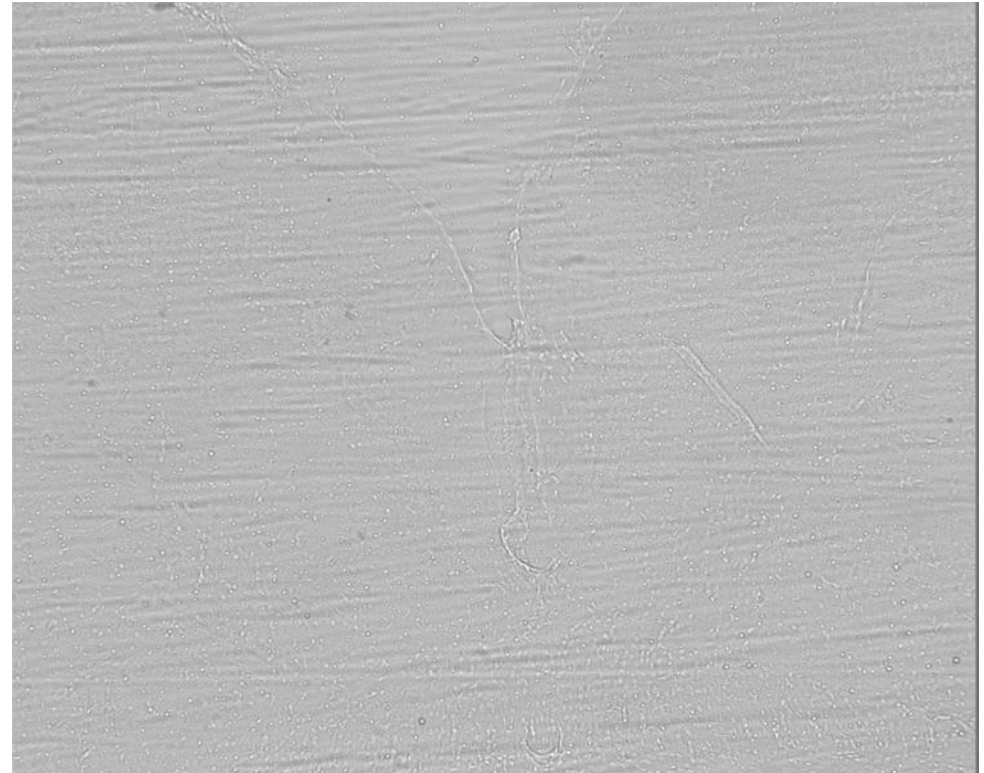

Supplement: Supplementary file 1 [file Image_1.pdf]
