# Supplementary material for: Disrupted development from head to tail: Pervasive effects of postnatal restricted resources on neurobiological, behavioral, and morphometric outcomes
Source: Front Behav Neurosci. 2022 Aug 5;16:910056. doi: 10.3389/fnbeh.2022.910056 (PMC9389412; doi:10.3389/fnbeh.2022.910056)
Supplement: Supplementary file 2 [file Table_1.pdf]

Table 1. Litter characteristics*

Mom ID	Resource Group	Number of Pups	Males	Females
**1**	Standard Resource	13	8	5
**2**	Standard Resource	11	6	5
**5**	Standard Resource	11	6	5
**6**	Low Resource	11	6	5
**7**	Low Resource	11	5	6
**8**	Standard Resource	10	4	6
**9**	Low Resource	13	8	5
**10**	Standard Resource	10	5	5
**11**	Low Resource	10	10	0
**12**	Standard Resource	12	7	5

*The number of male and female pups for each litter used in the study; note females #3 and #4 did not get pregnant and are not included in the list.
