# Supplementary material for: Disrupted development from head to tail: Pervasive effects of postnatal restricted resources on neurobiological, behavioral, and morphometric outcomes
Source: Front Behav Neurosci. 2022 Aug 5;16:910056. doi: 10.3389/fnbeh.2022.910056 (PMC9389412; doi:10.3389/fnbeh.2022.910056)
Supplement: Supplementary file 3 [file Table_2.pdf]

Table 2. Nonsignificant findings throughout the study.

Behavioral Data:

Behavioral Test	Dependent variable	Independent variable	Mean ± SEM
**Maternal responsiveness**	Total time to complete (sec)	Environment	Standard Resource = 308.5 ± 51.5<br>Low Resource = 303.02 ± 56.7
	Number of pups	Environment	Standard Resource = 3.0 ± 0.55<br>Low Resource= 2.75 ± 0.75
**Play behavior**	Play attack evasion 1 (frequency)	Sex	Female = 2.63 ± 0.63<br>Male = 2.6 ± 0.57
		Environment	Standard Resource = 2.93 ± 0.75<br>Low Resource= 2.31 ± 0.45
		Sex X Environment	Female Standard Resource = 2.75 ± 1.11<br>Female Low Resource= 2.5 ± 0.68<br>Male Standard Resource = 3.17 ± 1.05<br>Male Low Resource = 2.13 ± 0.64
	Play attack evasion 2	Sex	Female = 1.44 ± 0.398<br>Male = 2.71 ± 0.55
		Environment	Standard Resource = 2.07 ± 0.53<br>Low Resource= 2.0 ± 0.47
		Sex X Environment	Female Standard Resource = 1.63 ± 0.46<br>Female Low Resource= 1.25 ± 0.67<br>Male Standard Resource = 2.67 ± 1.1<br>Male Low Resource= 2.75 ± 0.59
	Play attack evasion 3	Sex	Female = 1.63 ± 0.0.35<br>Male = 1.5 ± 0.63
		Environment	Standard Resource = 1.64 ± 0.51<br>Low Resource = 1.5 ± 0.34
		Sex X Environment	Female Standard Resource = 1.5 ± 0.62<br>Female Low Resource= 1.75 ± 0.37<br>Male Standard Resource = 1.8 ± 0.91<br>Male Low Resource= 1.25 ± 0.59
	Pins 1 (frequency)	Sex	Female = 2.44 ± 0.55<br>Male = 2.43 ± 0.78
		Environment	Standard Resource = 3.07 ± 0.82<br>Low Resource= 1.88 ± 0.46
		Sex X Environment	Female Standard Resource = 2.75 ± 0.75<br>Female Low Resource = 2.1 ± 0.83<br>Male Standard Resource = 3.5 ± 1.7<br>Male Low Resource= 1.63 ± 0.42
	Pins 2	Sex	Female = 3.38 ± 0.77<br>Male = 4.14 ± 1.41
		Environment	Standard Resource = 3.71 ± 1.24<br>Low Resource = 3.75 ± 0.98
		Sex X Environment	Female Standard Resource = 3.13 ± 0.88<br>Female Low Resource= 3.63 ± 1.34<br>Male Standard Resource = 4.5 ± 2.78

			Male Low Resource = 3.88 ± 1.5
	Pins 3	Sex	Female = 3.94 ± 0.81<br>Male = 3.43 ± 1.06
		Environment	Standard Resource = 4.07 ± 1.13<br>Low Resource= 3.38 ± 0.73
		Sex X Environment	Female Standard Resource = 3.13 ± 1.27<br>Female Restricted Low = 4.75 ± 0.996<br>Male Standard Resource = 5.33± 2.04<br>Male Low Resource= 2.0 ± 0.87
**Social investigation assessment container**	Distance travelled (mm)	Sex	Female = 2553.12 ± 107.2<br>Male = 2529.59 ± 94.43
		Environment	Standard Resource = 2538.23 ± 109.4<br>Low Resource= 2545.55 ± 95.9
		Sex X Environment	Female Standard Resource = 2501.8 ± 180.96<br>Female Low Resource= 2604.5 ± 125.5<br>Male Standard Resource = 2586.8 ± 102.9<br>Male Low Resource = 2486.7 ± 150.66
	Interactions (frequency)	Sex	Female = 26.5 ± 3.22<br>Male = 25.0 ± 2.61
		Environment	Standard Resource = 29.36 ± 3.23<br>Low Resource= 22.69 ± 2.51
		Sex X Environment	Female Standard Resource = 29.0 ± 4.95<br>Female Low Resource = 24.0 ± 4.27<br>Male Standard Resource = 29.83 ± 4.21<br>Male Low Resource= 21.38 ± 2.89
	Rearing (frequency)	Sex	Female = 13.25 ± 0.92<br>Male = 11.58 ± 0.45
		Environment	Standard Resource = 12.79 ± 1.13<br>Low Resource= 12.44 ± 0.76
		Sex X Environment	Female Standard Resource = 13.13± 1.59<br>Female Low Resource = 13.38 ± 1.05<br>Male Standard Resource= 12.33 ± 1.71<br>Male Low Resource= 11.15 ± 1.05
	Grooming (frequency)	Sex	Female = 1.94 ± 0.34<br>Male = 2.29 ± 0.45
		Environment	Standard Resource = 2.21 ± 0.35<br>Low Resource= 2.0 ± 0.42
		Sex X Environment	Female Standard Resource = 1.88 ± 0.35<br>Female Low Resource= 2.0 ± 0.60<br>Male Standard Resource = 2.67 ± 0.67<br>Male Low Resource= 2.0 ± 0.42
**Social investigation assessment conspecific**	Distance traveled (mm)	Sex	Female = 2336.07 ± 111.54<br>Male = 2164.38 ± 89.95
		Environment	Standard Resource = 2188.09 ± 96.69<br>Low Resource = 2313.94 ± 108.73
		Sex X Environment	Female Standard Resource = 2302.11 ± 140.35<br>Female Low Resource = 2374.87 ± 188.345

			Male Standard Resource = 2036.05 ± 108.86<br>Male Low Resource= 2260.62 ± 129.85
	Interactions (frequency)	Sex	Female = 26.50 ± 3.37<br>Male = 32.64 ± 3.58
		Environment	Standard Resource = 27.07 ± 3.31<br>Low Resource= 31.38 ± 3.66
		Sex X Environment	Female Standard Resource = 23.13 ± 4.79<br>Female Low Resource= 29.88 ± 4.73<br>Male Standard Resource = 32.33 ± 3.70<br>Male Low Resource= 32.88 ± 5.86
	Duration interaction (sec)	Sex	Female = 48.89 ± 7.09<br>Male = 57.78 ± 6.05
		Environment	Standard Resource = 55.88 ± 7.91<br>Low Resource = 50.55 ± 5.67
		Sex X Environment	Female Standard Resource = 51.87 ± 12.95<br>Female Low Resource= 45.9 ± 6.73<br>Male Standard Resource = 61.23 ± 7.52<br>Male Low Resource= 55.19 ± 9.3
	Grooming (frequency)	Sex	Female = 2.0 ± 0.40<br>Male = 1.57 ± 0.20
		Environment	Standard Resource = 1.93 ± 0.30<br>Low Resource = 1.67 ± 0.35
		Sex X Environment	Female Standard Resource = 2.0 ± 0.46<br>Female Low Resource= 2.0 ± 0.72<br>Male Standard Resource = 1.83 ± 0.40<br>Male Low Resource= 1.38 ± 0.18
**Open Field Task**	Duration freezing (sec)	Sex	Female = 91.25 ± 3.30<br>Male = 111.63 ± 14.04
		Environment	Standard Resource = 94.36 ± 6.87<br>Low Resource= 106.36 ± 11.54
		Sex X Environment	Female Standard = 91.26 ± 4.28<br>Female Low Resource= 91.24 ± 5.33<br>Male Standard Resource = 98.48 ± 15.68<br>Male Low Resource= 121.49 ± 21.84
	Crossing center (frequency)	Sex	Female = 4.0 ± 0.58<br>Male = 4.79 ± 0.58
		Environment	Standard Resource = 5.07 ± 0.65<br>Low Resource= 3.75 ± 0.49
		Sex X Environment	Female Standard Resource = 4.25 ± 0.94<br>Female Low Resource = 3.75 ± 0.75<br>Male Standard Resource = 6.17 ± 0.70<br>Male Low Resource= 3.75 ± 0.67
	Rearing (frequency)	Sex	Female = 17.63 ± 1.12<br>Male = 18.07 ± 0.96
		Environment	Standard Resource = 16.71 ± 1.05<br>Low Resource= 18.8 ± 0.99
		Sex X Environment	Female Standard Resource = 16.25 ± 1.71

			Female Low Resource= 19.0 ± 1.38<br>Male Standard Resource = 17.33 ± 1.05<br>Male Low Resource = 18.63 ± 1.52
	Thigmotaxis (frequency)	Sex	Female = 39.0 ± 3.68<br>Male = 30.79 ± 4.7
		Environment	Standard Resource = 35.0 ± 4.36<br>Low Resource = 35.31 ± 4.24
		Sex X Environment	Female Standard Resource = 36.38 ± 6.13<br>Female Low Resource= 41.63 ± 4.29<br>Male Standard Resource = 33.17 ± 6.62<br>Male Low Resource = 29.0± 6.88
	Distance traveled (mm)	Sex	Female = 3794.13 ± 248.15<br>Male = 2998.94 ± 321.25
		Environment	Standard Resource = 3648.93 ± 260.59<br>Low Resource = 3225.39 ± 320.05
		Sex X Environment	Female Standard Resource = 3862.88 ± 404.21<br>Female Low Resource= 3725.38 ± 314.89<br>Male Standard Resource = 3363.66 ± 280.15<br>Male Low Resource= 2725.40 ± 518.1
**Dry land maze Training and acquisition**	Latency to first baited well habituation 1 (sec)	Sex	Female = 343.31 ± 9.11<br>Male = 337.79 ± 11.87
		Environment	Standard Resource = 355.14 ± 3.33<br>Low Resource = 328.13 ± 12.64
		Sex X Environment	Female Standard Resource = 351.5 ± 5.62<br>Female Low Resource = 335.13 ± 17.47<br>Male Standard Resource = 360.0 ± 0<br>Male Low Resource = 321.13 ± 19.11
	Latency to first baited well habituation 2	Sex	Female = 183.53 ± 29.88<br>Male = 183.87 ± 27.23
		Environment	Standard Resource = 210.09 ± 31.07<br>Low Resource= 160.59 ± 25.44
		Sex X Environment	Female Standard Resource = 212.0 ± 46.47<br>Female Low Resource = 155.05 ± 37.89<br>Male Standard Resource = 207.53 ± 42.7<br>Male Low Resource = 166.13 ± 36.45
	Latency to first baited well habituation 3	Sex	Female = 179.54 ± 22.83<br>Male = 156.36 ± 24.28
		Environment	Standard Resource = 146.7 ± 28.71<br>Restricted Low = 188.03 ± 17.51
		Sex X Environment	Female Standard Resource = 145.2 ± 38.68<br>Female Low Resource = 213.88 ± 20.02<br>Male Standard Resource = 148.6 ± 46.97<br>Male Low Resource= 162.18 ± 26.87
	Latency to first baited well Acquisition	Sex	Female = 100.99 ± 22.97<br>Male = 59.39 ± 12.38
		Environment	Standard Resource = 69.63 ± 20.65<br>Low Resource = 92.03 ± 18.92

		Sex X Environment	Female Standard Resource = 84.88 ± 35.11<br>Female Low Resource = 117.1 ± 30.88<br>Male Standard Resource = 49.3 ± 11.87<br>Male Low Resource= 66.95 ± 20.08
**Dry land maze testing**	Latency to well Test 1	Sex	Female = 40.57 ± 3.83<br>Male = 60.49 ± 10.36
		Environment	Standard Resource = 39.34 ± 6.07<br>Low Resource= 57.49 ± 7.81
		Sex X Environment	Female Standard Resource = 31.84 ± 4.4<br>Female Low Resource = 48.22 ± 4.74<br>Male Standard Resource = 49.85 ± 12.46<br>Male Low Resource= 68.09 ± 15.49
	Latency to well Test 2	Sex	Female = 41.33 ± 8.21<br>Male = 34.64 ± 5.25
		Environment	Standard Resource = 39.03 ± 8.6<br>Low Resource= 37.82 ± 6.3
		Sex X Environment	Female Standard Resource = 47.57 ± 14.09<br>Female Low Resource= 35.87 ± 9.74<br>Male Standard Resource = 27.08 ± 3.18<br>Male Low Resource = 40.04 ± 8.38
	Latency to well Test 3	Sex	Female = 45.83 ± 6.61<br>Male = 52.13 ± 10.33
		Environment	Standard Resource = 51.45 ± 8.51<br>Low Resource= 46.37 ± 8.1
		Sex X Environment	Female Standard Resource = 55.18 ± 10.76<br>Female Low Resource= 37.65 ± 7.51<br>Male Standard Resource = 46.24 ± 14.95<br>Male Low Resource = 56.33 ± 14.89
	Errors Test 1 (frequency)	Sex	Female = 0.98 ± 0.17<br>Male = 0.76 ± 0.22
		Environment	Standard Resource = 0.76 ± 0.19<br>Low Resource = 0.98 ± 0.19
		Sex X Environment	Female Standard Resource = 0.92 ± 0.29<br>Female Low Resource = 1.04 ± 0.18<br>Male Standard Resource = 0.56 ± 0.22<br>Male Low Resource= 0.92 ± 0.34
	Errors Test 2	Sex	Female = 0.88 ± 0.16<br>Male = 0.81 ± 0.14
		Environment	Standard Resource = 0.93 ± 0.16<br>Low Resource= 0.77 ± 0.14
		Sex X Environment	Female Standard Resource = 1.04 ± 0.23<br>Female Low Resource = 0.71 ± 0.23<br>Male Standard Resource = 0.78 ± 0.24<br>Male Low Resource= 0.83 ± 0.18
	Errors Test 3	Sex	Female = 0.65 ± 0.14<br>Male = 1.17 ± 0.21
		Environment	Standard Resource = 0.74 ± 0.18

			Low Resource= 1.02± 0.19
		Sex X Environment	Female Standard Resource = 0.71 ± 0.27<br>Female Low Resource = 0.58 ± 0.12<br>Male Standard Resource = 0.78 ± 0.24<br>Male Low Resource= 1.46 ± 0.29
	Fecal boluses Test 1 (number)	Sex	Female = 0.19 ± 0.08<br>Male = 0.52 ± 0.21
		Environment	Standard Standard = 0.21 ± 0.09<br>Low Resource= 0.46± 0.19
		Sex X Environment	Female Standard Resource = 0.21 ± 0.13<br>Female Low Resource= 0.17 ± 0.11<br>Male Standard Resource = 0.22 ± 0.14<br>Male Low Resource = 0.75± 0.34
	Fecal boluses Test 2	Sex	Female = 0.13 ± 0.09<br>Male = 0.29 ± 0.098
		Environment	Standard Resource = 0.24 ± 0.11<br>Low Resource= 0.17 ± 0.08
		Sex X Environment	Female Standard Resource = 0.25 ± 0.16<br>Female Low Resource= 0.0 ± 0.0<br>Male Standard Resource = 0.22 ± 0.14<br>Male Low Resource = 0.33± 0.14
	Fecal boluses Test 3	Sex	Female = 0.23 ± 0.13<br>Male = 0.26 ± 0.12
		Environment	Standard Resource = 0.26 ± 0.12<br>Low Resource = 0.23 ± 0.11
		Sex X Environment	Female Standard Resource = 0.38 ± 0.25<br>Female Low Resource = 0.08 ± 0.08<br>Male Standard Resource = 0.11 ± 0.11<br>Male Low Resource= 0.38 ± 0.19
	Time to Completion Test 1 (sec)	Sex	Female = 57.8 ± 4.64<br>Male = 67.7 ± 9.51
		Environment	Standard Resource = 61.11 ± 6.6<br>Low Resource= 63.6 ± 7.72
		Sex X Environment	Female Standard Resource = 59.73 ± 8.23<br>Female Low = 55.95 ± 4.82<br>Male Standard Resource = 62.96 ± 11.77<br>Male Low Resource = 71.28 ± 14.68
	Time to Completion Test 2 (sec)	Sex	Female = 51.02 ± 8.14<br>Male = 45.28 ± 5.61
		Environment	Standard Resource = 47.33 ± 7.92<br>Low Resource = 49.22 ± 6.59
		Sex X Environment	Female Standard Resource = 56.23 ± 13.09<br>Female Low Resource= 45.81 ± 10.23<br>Male Standard Resource = 35.48 ± 3.4<br>Male Low Resource = 52.63 ± 8.85
	Time to Completion Test 3	Sex	Female = 54.17 ± 7.07<br>Male = 66.47 ± 13.17

	(sec)	Environment	Standard Resource = 56.84 ± 8.34<br>Restricted Low = 62.6± 11.51
		Sex X Environment	Female Standard Resource = 63.01 ± 11.6<br>Female Low Resource= 79.86 ± 20.62<br>Male Standard Resource = 48.62 ± 12.12<br>Male Low Resource= 79.86 ± 20.62
**Dry land maze probe**	Internal rear (frequency)	Sex	Female = 0.71 ± 0.3<br>Male = 1.43 ± 0.51
		Environment	Standard Resource = 1.14 ± 0.38<br>Low Resource = 1.0 ± 0.48
		Sex X Environment	Female Standard Resource = 0.5 ± 0.27<br>Female Low Resource= 1.0 ± 0.63<br>Male Standard Resource = 2.0 ± 0.68<br>Male Low Resource = 1.0 ± 0.73

Endocrine Data:

**Dependent variable**	Independent variable	Mean ± SEM
**CORT (pg/ml)**	Sex	Female = 526.18 ± 107.95<br>Male = 624.06 ± 196.49
	Environment	Standard Resource = 627.17± 161.54<br>Low Resource = 523.27 ± 149.86
	Sex X Environment	Female Standard = 752.89 ± 260.84<br>Female Low Resource= 267.07 ± 32.66<br>Male Standard Resource= 459.56 ± 312.67<br>Male Low Resource= 747.44 ± 260.84
**DHEA/CORT Ratio**	Sex	Female = 3.18 ± 1.24<br>Male = 2.6 ± 0.73
	Environment	Standard Resource= 1.45 ± 0.28<br>Low Resource= 4.25 ± 1.29
	Sex X Environment	Female Standard Resource = 1.41 ± 0.3<br>Female Low Resource= 5.21 ± 2.5<br>Male Standard Resource = 1.52 ± 0.54<br>Male Low Resource= 3.4 ± 1.24

Micro-computed tomography (micro-CT) data:

**Dependent variable**	Independent variable	Mean ± SEM
**Trabecular thickness**	Sex	Female = 0.46 ± 0.008<br>Male = 0.44 ± 0.005
	Environment	Standard Resource = 0.45 ± 0.007<br>Low Resource= 0.45 ± 0.007
	Sex X Environment	Female Standard Resource = 0.46 ± 0.0095<br>Female Low Resource= 0.46 ± 0.014<br>Male Standard Resource = 0.45 ± 0.0092<br>Male Low Resource= 0.44 ± 0.0075

Neuroquantification Data:

**Immunoreactivity**	Brain area	Independent variable	Mean $\pm$ SEM
**c-Ffos**<br>(number of cells)	CA3	Sex	Female = 64.58 ± 1.27<br>Male = 63.73 ± 1.22
		Environment	Standard Resource = 64.62 ± 1.64<br>Low Resource= 63.8 ± 0.84
		Sex X Environment	Female Standard Resource = 65.73 ± 2.04<br>Female Low Resource= 63.44 ± 1.54<br>Male Standard Resource = 63.15 ± 2.8<br>Male Low Resource= 64.17 ± 0.78
**Glucocorticoid Receptor-IR**<br>(number of cells)	BLA	Sex	Female = 77.73 ± 3.7<br>Male = 70.04 ± 3.47
		Environment	Standard Resource = 71.43 ± 4.36<br>Low Resource = 76.52 ± 3.07
		Sex X Environment	Female Standard Resource = 73.28 ± 5.78<br>Female Low Resource = 82.19 ± 4.44<br>Male Standard Resource = 68.98 ± 7.15<br>Male Low Resource= 70.84 ± 3.4
**Iba1-IR (for Microglia detection)**<br>(number of cells**)**	BLA	Sex	Female = 0.03 ± 0.0027<br>Male = 0.034 ± 0.0032
		Environment	Standard Resource = 0.032 ± 0.0037<br>Low Resource 0.032 ± 0.0023
		Sex X Environment	Female Standard Resource = 0.034 ± 0.0048<br>Female Low Resource = 0.027 ± 0.0023<br>Male Standard Resource = 0.029 ± 0.0059<br>Male Low Resource= 0.037 ± 0.0031
